# The role of biomaterials in burn treatment

**DOI:** 10.4103/2321-3868.143608

**Published:** 2014-10-25

**Authors:** Luigi Ambrosio

**Affiliations:** Department of Chemical Science and Materials Technology, National Research Council, 00185 Rome, Italy

Burns are among the most challenging injuries to manage. Although the great advances in treatment that have been made have arguably been sufficient to address the related medical challenges of the past century, modern society now faces even greater difficulties. These are caused predominantly by increase in population and lifespan, which frequently are not matched by an increased effectiveness in the maintenance of health and quality of life.

Because aging people are more prone to illness, keeping people healthy and active, while managing healthcare costs, is an important societal challenge. The ethical and economic dilemma is how to achieve equality in quality of care while simultaneously decreasing the cost of care for an ever-expanding number of people.

Burn injuries, leading to significant mortality and morbidity all over the word, are among the most devastating of all injuries, with outcomes varying from physical impairments and disabilities to emotional and mental consequences. The management of burns and their sequelee, even in the well-equipped, modern burn units of advanced and affluent societies, remains demanding and extremely costly.[1]

Consequently, considerable effort is needed to define an appropriate strategy for the successful implementation of therapeutic treatments in terms of performance and cost. In the context of these complex needs, biomaterials and related novel technologies may play an important role because it is possible to precisely customize their overall properties.

Generally, burns cause significant fluid loss and extensive tissue damage resulting from deep wounds by impairing many of the vital functions performed by the skin. Wound infection, which further exacerbates the local tissue damage, is a common complication, and systemic inflammatory and immunological responses can arise that may lead to a higher predisposition toward life-threatening sepsis and multi-organ failure.[2] In such cases, easy and appropriate clinical treatments are critical to reduce the mortality rates associated with the injury.

**Table 1:** 

Access this article online	
**Quick Response Code**:	**Website**: www.burnstrauma.com
	**DOI**: 10.4103/2321-3868.143608

These considerations suggest an expanded emphasis on the following to satisfy the growing need for effective burn treatment:

1. Regeneration of tissues, and
2. Prevention of tissue deterioration.

This note outlines the clinical needs that could be met by innovative bioactive materials in the near future, including the repair and regeneration of soft tissues and the rapid, low-cost analysis of human cell-biomaterial interactions leading to patient-specific diagnoses and treatments using molecularly tailored biomaterials.

Various therapies that effect wound repair have been proposed over the past few decades. The innovative application of bioactive materials with genetic control of *in situ* tissue responses offers the potential to achieve both tissue regeneration and prevention of tissue deterioration. The clinical success of the use of bioactive biomaterials for regeneration provides evidence that this approach is effective; likewise, there is evidence that the prevention of tissue deterioration is also possible.

Bioengineered products that can facilitate the healing of chronic ulcers, deep wounds, and burns are in increased demand to improve patients’ quality of life and survival rate. Many different biomaterials have been investigated for the use as dermal substitutes[3] and no material has yet been described that provides fulfils all the ideal features for such a purpose.

The clinical performance of wound dressings and dermal substitutes based on synthetic biomaterials is limited by the ability of these polymers to keep the wound environment moist, to protect the wound site from infections, and, in some cases, to encourage cell colonization.[4,5] Natural polymers of human and animal origin exhibit biochemical features that can facilitate the healing process. Indeed, biomaterials based on protein or polysaccharide compositions, such as collagen, glycosaminoglycans, agarose, alginate, chitosan, and fibrin glue, have been shown to be able to promote interactions with biochemical and cellular components, leading, for example, to hemostatic functions (*e.g.*, alginate and fibrin) and to bioligand-driven cell-activity control (*e.g.*, collagen and hyaluronan).[6] In this issue, the article by Longinotti discusses the role of hyaluronic acid in skin tissue formation and wound healing, and the use of hyaluronic acid (HA) based dressings and dermal substitutes is reviewed, especially the successful clinical application of HYAFF® film, a commercial medical device.[7] Natural polymers from plants and marine organism, such as vegetable proteins, cellulose, chitosan, *etc.*, have also been studied as wound dressing materials. The article entitled “Pre-clinical evaluation of soybean-based wound dressings and dermal substitute formulations in pig healing and non-healing *in vivo* models” by Shevchenko and Santin, demonstrates the function of such materials in wound healing and the importance of choosing optimized formulations for different situations.[8] However, the use of biopolymers in regenerative medicine is limited by their antigenic potential, by the risk of transmittable diseases, and, in some cases, by their relatively high extraction costs.

Initially, raw human or animal skin, either fresh or frozen, was used to cover wounds but immunological reactions were soon observed leading to poor experimental and clinical performances. These demonstrated no rejection upon implantation while providing a solid dermal wound bed for the later application of split-thickness skin grafts.[9] Although this approach has been clinically successful, it still suffers certain drawbacks that must be resolved.

A class of therapies is emerging that is based on gene and stem cell therapy. Gene therapy, which was initially developed to treat congenital defects, is a newly viable option for the enhancement of wound repair. Gene encoding for growth factors or cytokines has shown the greatest potential in accelerating wound closure. Most of the gene-delivery systems are on the basis of naked DNA application, high-pressure injection, viral transfection, or liposomal vectors. Stem cell therapy is based on the prolonged capacity of embryonic and adult stem cells that can differentiate into various tissue types. Stem cells isolated from various sources, including bone marrow, umbilical cord blood, peripheral blood, adipose tissue, skin, and hair follicles, have been used to accelerate the wound healing process of acute and chronic wounds.[10]

In this issue, Wang *et al.* introduce the use of collagen-binding MSC affinity peptide as a scaffold in treating acute full-thickness skin wounds *in vivo*. The scaffold demonstrated the ability to capture MSCs to injury sites and to accelerate wound healing.[11] Recently, the combination of gene and stem cell therapy has become a promising way to treat acute and chronic wounds, though it remains at an experimental level.

The engineering of dermal substitutes and their application in human patients has come into reality.[7] However, surgeons, materials scientists, biochemists and biologists, are still striving to develop complex biomimetic skin substitutes which can be readily used in large quantities, possibly in only one surgical intervention and without obvious scarring. The ambitious goal is now to construct a dermoepidermal substitute that can get rapid vascularization, optimally supports a stratifying epidermal graft on a biodegradable matrix, and can be easily handled by the surgeon. After all, this goal must be achieved in compliance with both strict safety requirements and the harsh realities of the economic market.[12]

Nanotechnologies and nanomaterials have introduced new therapeutic horizons in medical research, and innovative technologies are rapidly advancing for the development of novel bioactive materials for burn-wound care that are able to mimic the extracellular matrix, including its structure, biological cues, cells, and biofunctionality. Moreover, efforts in the development of novel strategies for modifying the nanotopography of surfaces have recently been applied to the fabrication of new generations of bactericidal biomaterials through surface coatings or surface-chemical modifications.[13]

Hydrogels/gels and related composites appear to be optimal biomaterials for burn management that employ both regenerative and reparative strategies. Because of their intrinsic structure, hydrogels and gels prevent the presence of excessive exudates in the wound while also, contributing to the digestion of eschar and necrotic tissue. They enable the following:

1. The inclusion of nanofibers to fulfill structural needs;

2. The inclusion of nano- and/or microspheres to include biological cues, for both regenerative and therapeutic needs, to induce delivery on demand;

3. The inclusion of specific cells for optimal regeneration;

4. The modulation of the nano- and microporosity of the materials to promote angiogenesis and reinnervation;

5. Surface-chemical modification to achieve anti-adhesive and antimicrobial effects; and

6. Biodegradability. The use of polymeric hydrogels as advanced skin wound dressings and regenerative templates in wound care is reviewed by Madaghiel *et al.* in this issue. This review contributes to the understanding of the functions of polymeric hydrogels, as well as the research and application of hydrogels for wound healing.[14]

## Conclusions

There is considerable potential for hydrogels and polymer-based materials to facilitate the achievement of better treatment for burn injuries without neglecting healthcare needs. However, for the implementation of this approach, several aspects should be considered:

1. Research and product development should be directed toward specific clinical needs;
2. A knowledge systemic approach should be taken, fostered by the integration of various disciplines based on key enabling technologies; and
3. Appropriate actions should always consider the mental, social, and physical health of the patient.

## Call for Papers!

Burns & Trauma, an Open Access journal focusing on latest and best achievements in basic, clinical and translational research related to burns and trauma, warmly invited you to submit manuscripts on the upcoming special issues:

- Regeneration
- Metabolism & Nutrition
- Scar management & Tissue engineering

We guarantee timely response to submissions within two weeks.

Submit your paper: http://www.journalonweb.com/bt
